# Capecitabine-Induced Ileitis: A Case Report and Review of the Literature

**DOI:** 10.7759/cureus.91662

**Published:** 2025-09-05

**Authors:** Hanna Mohammed Sherief, Anita Nagetey, Muhammad Fahad, Qurrat Ul Ain Tahir

**Affiliations:** 1 Oncology, University Hospital Coventry and Warwickshire, Coventry, GBR; 2 Medicine, Sapthagiri Institute of Medical Sciences and Research Centre, Bengaluru, IND; 3 Internal Medicine, University Hospital Coventry and Warwickshire, Coventry, GBR

**Keywords:** capecitabine, capecitabine-induced diarrhoea, capecitabine side effects, fluoropyrimidine, ileitis, terminal ileitis

## Abstract

Capecitabine-induced ileitis is a rare but important complication, and awareness of this entity is crucial for oncologists and gastroenterologists to ensure timely recognition, appropriate management, and to avoid unnecessary interventions. We present the case of a 62-year-old woman with recurrent estrogen receptor (ER)-positive, human epidermal growth factor receptor 2 (HER2)-negative left breast cancer who developed vomiting and diarrhea following an increased dose of capecitabine. She was managed conservatively with intravenous fluids, antibiotics, and antiemetics. Imaging suggested findings consistent with ileitis. She was advised to rest her bowel and gradually reintroduce oral intake as tolerated, while receiving symptom relief with antiemetics and loperamide for diarrhea. Her blood parameters were closely monitored, and electrolyte imbalances were corrected as needed. With symptomatic improvement, she was discharged and subsequently reviewed in the clinic. Based on the most recent imaging and evidence of disease progression, a new treatment regimen of exemestane combined with everolimus was initiated.

## Introduction

Capecitabine, an oral fluoropyrimidine prodrug, is a valuable alternative to intravenous 5-fluorouracil (5-FU) in the treatment of colon and metastatic breast cancers. It has been associated with a lower incidence of adverse effects, including diarrhea (47.7% vs. 58.2%), neutropenia, and stomatitis, compared to IV 5-FU. These benefits have contributed to its increased adoption in clinical practice [1,2]. Capecitabine-induced ileitis is an uncommon adverse effect, with only a limited number of case reports documented in the literature. The drug undergoes enzymatic conversion to 5-FU, primarily in tumor tissues but also in normal gastrointestinal mucosa.

Following oral administration at a dose of 1250 mg/m^2^, capecitabine is rapidly and efficiently absorbed from the gastrointestinal tract, reaching its peak plasma concentration (Cmax) of 3 to 4 mg/L within approximately two hours (tmax). It has a relatively short elimination half-life, ranging from 0.55 to 0.89 hours. Nearly all drug-related substances are recovered in the urine and feces, indicating nearly 100% excretion [3].

This localized conversion may contribute to mucosal inflammation and injury, explaining the development of ileitis in susceptible individuals [4]. Gastrointestinal side effects occur more frequently than systemic ones with capecitabine and typically include nausea, vomiting, abdominal pain, mouth sores, and diarrhea [1,2]. Rarely, capecitabine has been implicated in causing segmental enteritis, particularly ileitis, which can mimic infectious or inflammatory bowel disease and delay appropriate management [4,5]. Recognizing this rare toxicity is crucial for clinicians, as early diagnosis and drug cessation are key to preventing complications. In this report, we describe a rare case of capecitabine-induced ileitis, highlighting its clinical presentation, diagnostic challenges, and management [6]. This discussion will focus on a clinical case involving capecitabine-induced ileitis and the subsequent approach to its management.

## Case presentation

The patient underwent a left mastectomy with level 2 axillary clearance in 2015. Adjuvant chemotherapy was administered using the FEC-T regimen, consisting of four cycles of fluorouracil, epirubicin, and cyclophosphamide (FEC) followed by four cycles of docetaxel. The patient also received adjuvant radiotherapy.

The patient was initially started on adjuvant letrozole but was switched to tamoxifen due to joint pain (arthralgia). The treatment plan included first-line palliative treatment with oral palbociclib and letrozole, along with denosumab. However, persistent pancytopenia was observed, even before initiating chemotherapy. Hematology assessment suggested that this was due to bone marrow infiltration from breast cancer. In 2024, imaging revealed progression of liver metastases on CT, accompanied by a rise in cancer antigen 15-3 (CA 15-3) levels. As a result, it was agreed to start capecitabine chemotherapy. The patient was found to have a dihydropyrimidine dehydrogenase (DPYD) mutation (was tested for DPYD 2A, 13, HapB3, and D949D, as is routinely done in the National Health Service (NHS)), consented to begin capecitabine at a reduced dose of 50% and was instructed to take two 500 mg tablets twice daily. However, during cycle 1, she developed nausea and vomiting after 14 days of treatment. Although advised to take 1000 mg twice daily, she had only been taking one 500 mg tablet twice a day, totalling 500 mg per dose. Prior to starting cycle 4, she was advised to increase the dose to 1000 mg twice daily.

**Figure 1:**
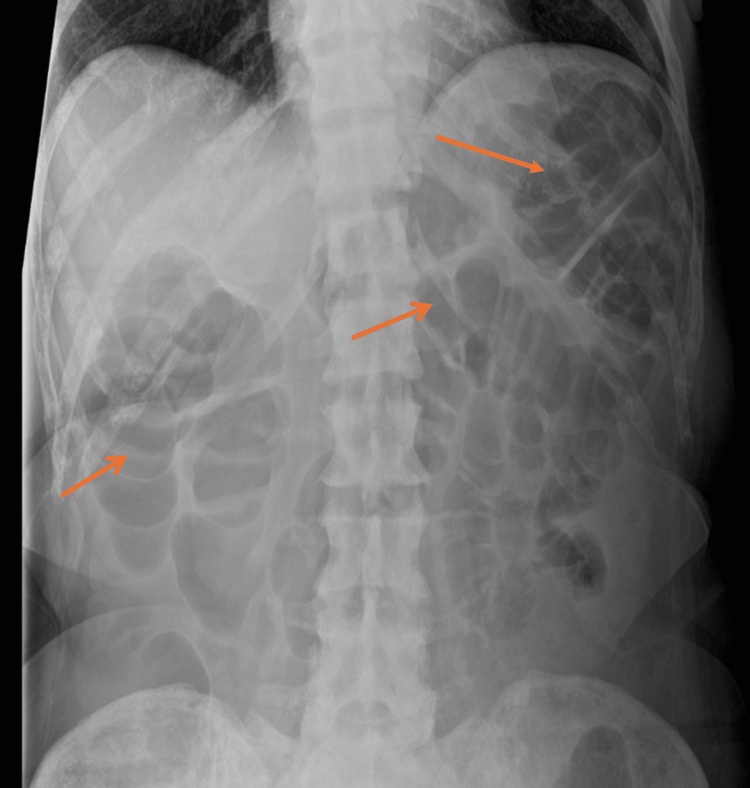
Abdominal X-ray showing prominent bowel loops without significant dilatation (arrows).

The patient presented with vomiting and diarrhoea following an increase in the dose of capecitabine. The patient had severe watery, non-bloody, non-mucoid diarrhoea, opening her bowels more than 10 times per day, associated with several episodes of vomiting, and was unable to keep any food or drink down. She was initiated on intravenous fluids, intravenous antibiotics (co-amoxiclav), and antiemetic therapy. Stool cultures did not show any infectious growth, and loperamide was commenced. She was advised to maintain hydration with liquids and to observe bowel rest. Abdominal radiography demonstrated prominent bowel loops without significant dilatation (Figure 1), leading to the discontinuation of loperamide due to suspicion of bowel obstruction versus capecitabine-induced colitis.

**Figure 2:**
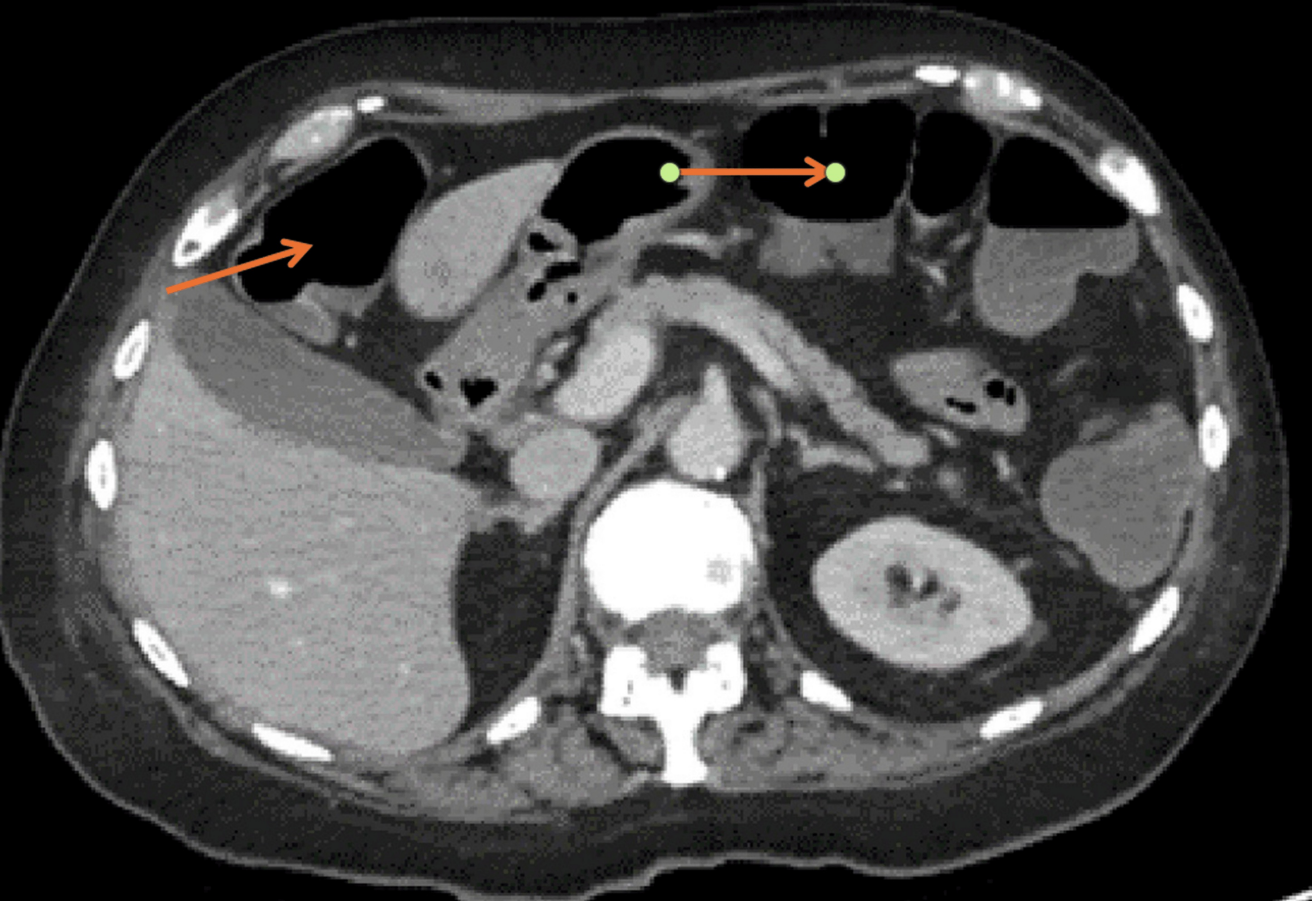
CT abdomen axial view showing bowel dilatation (arrows).

**Figure 3:**
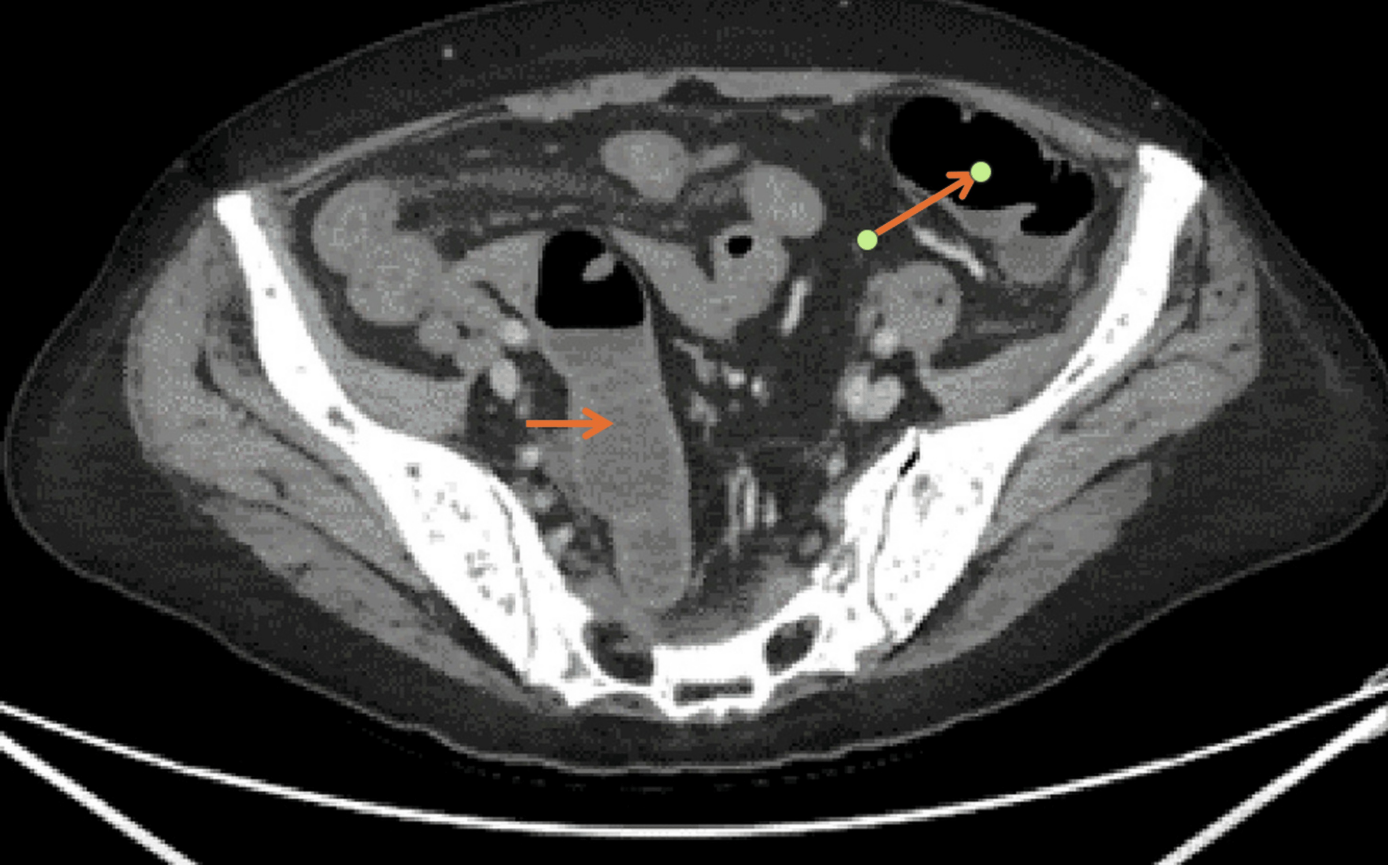
CT abdomen showing trace amounts of free pelvic fluid without evidence of drainable collections (arrows).

**Figure 4:**
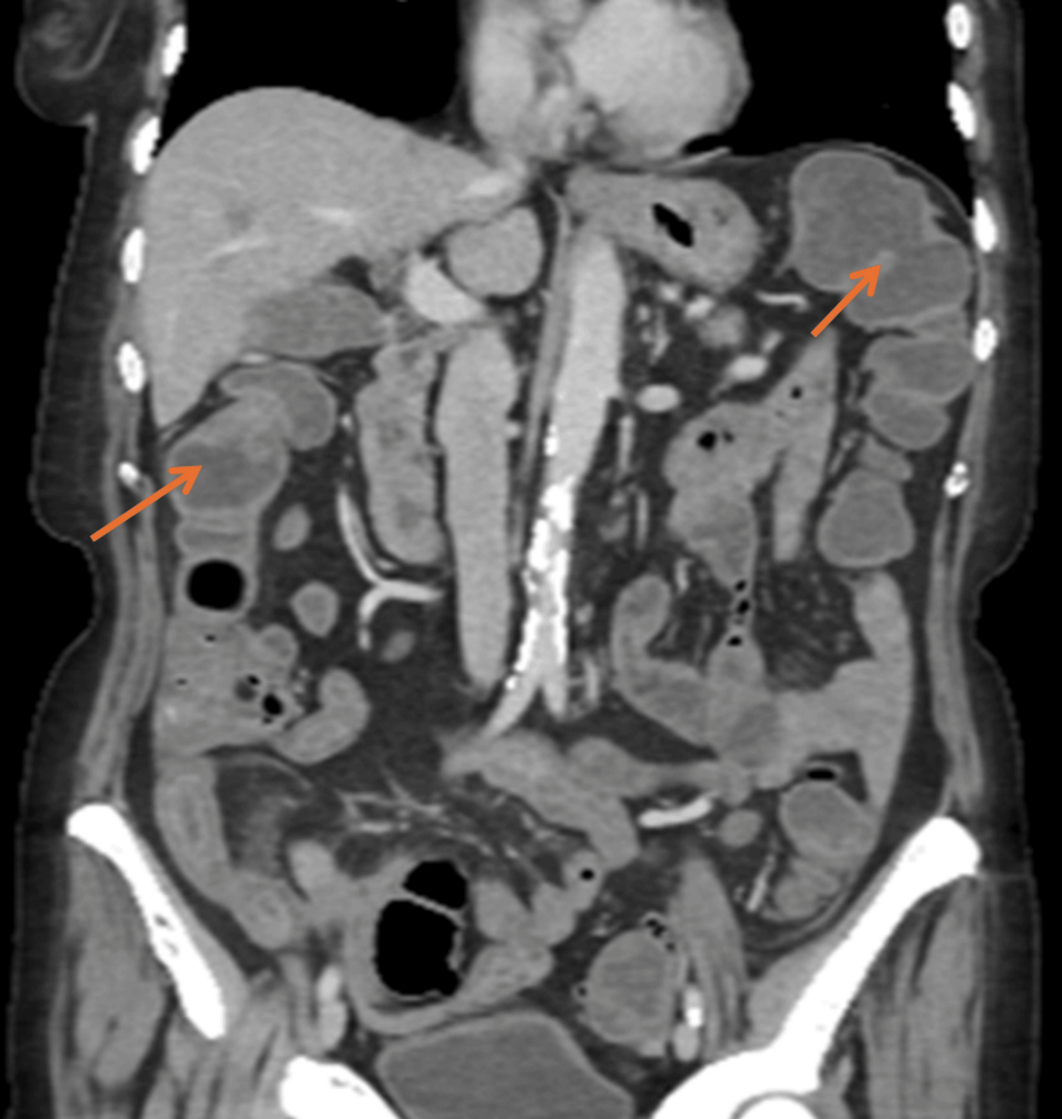
Coronal CT abdomen showing a long segment of mural thickening in the distal ileum (arrows).

Subsequently, a CT scan of the thorax, abdomen, and pelvis revealed a long segment of mural thickening in the distal ileum with associated mesenteric hyperaemia and vascular congestion extending to the ileocecal junction, indicative of distal ileitis likely secondary to chemotherapy. This ruled out bowel ischaemia. Trace amounts of free pelvic fluid were noted without evidence of drainable collections or bowel perforation (Figures 2-4).

​​​​​​Gastroenterology consultation recommended the reinstatement of loperamide, given the absence of infection or perforation, with an emphasis on conservative management. A review of the chemotherapy regimen was planned for a later date.

Electrolyte levels were monitored closely and corrected as necessary, particularly hypokalaemia and hypomagnesemia. Initial laboratory investigations revealed mild hypokalaemia, which was managed with intravenous potassium chloride. During the hospital stay, the patient developed hyperkalaemia (6.0 mmol/L), presumed iatrogenic, which subsequently resolved spontaneously by the following blood test (4.7 mmol/L). Hypomagnesemia was corrected with a single intravenous magnesium infusion, and hypophosphatemia was treated with oral supplementation.

CRP was initially high (61mg/L), which started to show a decreasing trend by the end of one week and was 10mg/L after two weeks. By this time, the patient’s clinical condition had improved, and she expressed a desire for discharge. Since infection was ruled out, this downward trend of CRP was likely secondary to inflammation.

Conservative management consisted of bowel rest, intravenous fluid therapy initially, followed by gradual reintroduction of oral fluids and nutrition, alongside symptomatic control with antiemetics and antidiarrheal agents, specifically loperamide. These interventions resulted in clinical improvement, and in two weeks, the patient was deemed fit for discharge.

A prior CT scan prior to this event demonstrated disease progression with an increase in the size and number of hepatic lesions. Following hospital discharge, the patient was reviewed in the clinic, and after discussion of therapeutic options, the patient commenced treatment with exemestane and everolimus, having provided informed consent.

## Discussion

Diarrhoea is a serious and potentially life-threatening complication commonly associated with many chemotherapy drugs [7]. Gastrointestinal side effects, particularly diarrhoea, are frequently triggered by anti-cancer therapies such as chemotherapy or radiation. Capecitabine is a widely used, orally administered chemotherapeutic agent known for causing gastrointestinal side effects. It may induce diarrhoea by damaging the intestinal mucosa [8]. Novel approaches, such as the use of probiotics or glutamine supplementation, are being explored to reduce mucosal damage, but further research is needed to establish their efficacy [9].

The relationship between dose escalation and DPYD mutations highlights a delicate balance - higher doses can improve efficacy but significantly raise toxicity risk in DPYD-deficient patients. Genotype-guided dosing remains critical to minimize severe adverse effects while maintaining therapeutic benefit. Genetic predisposition plays a critical role in susceptibility to fluoropyrimidine toxicity. Deficiency or mutations in the DPYD gene, responsible for the catabolism of 5-FU, lead to accumulation of active drug metabolites and an increased risk of severe adverse events, including diarrhoea and ileitis [10]. This relationship underscores the importance of genotype-guided dosing to balance efficacy with safety, minimizing severe toxicities in DPYD-deficient patients [11,12].

Capecitabine is linked to various side effects, including mucositis, hand-foot syndrome, nausea, vomiting, and fatigue. More severe complications, such as bowel obstruction and bowel perforation, have also been reported [13]. In this case, the patient tolerated a lower dose of capecitabine until cycle 3. However, when the dose was increased in cycle 4, she developed diarrhoea and vomiting, which were managed with bowel rest and anti-diarrhoeal therapy. This toxicity appeared to be dose-dependent. Such dose dependency has been reported in multiple studies, highlighting the need for cautious dose adjustments and close monitoring, especially during early treatment cycles [14,15].

Medications such as loperamide and somatostatin analogues can help control excessive bowel movements, although they may lead to additional adverse effects. Therefore, there is a need for simple and safe strategies to mitigate chemotherapy-induced diarrhoea, like a low threshold for diagnosis, early recognition, prophylactic loperamide, dose adjustments, or regular genetic screening (DPYD). Additionally, the method of chemotherapy administration can influence the severity of treatment-related toxicity, such as using radiotherapy with capecitabine [16]. This highlights the need for careful treatment planning and multidisciplinary coordination to mitigate overlapping toxicities. Preventative strategies include regular genetic screening for DPYD mutations, early recognition of symptoms, prophylactic use of anti-diarrhoeal agents in high-risk patients, and individualized dose adjustments [17].

As disease progression was noted on imaging, the decision was made to discontinue capecitabine and initiate treatment with exemestane and everolimus. In this case, discontinuation of capecitabine and initiation of exemestane with everolimus provided an effective alternative therapeutic approach following disease progression and intolerance to chemotherapy. This underscores the importance of balancing treatment efficacy and patient safety in oncologic care [18].

## Conclusions

The case report emphasizes that ileitis can arise despite prior good tolerance to capecitabine. Maintaining a high level of suspicion is essential for timely diagnosis. Early detection, careful monitoring, and appropriate supportive care can lead to the reversal of ileitis. The incidence of drug-induced ileitis could rise in the future, particularly due to the introduction of new medications, notably in immunology and oncology, with the emergence of innovative treatments.

Importantly, this case also underlines the dose-dependent nature of capecitabine toxicity, reinforcing the need for personalized dose adjustments, particularly in patients with known genetic variations such as DPYD mutations. It also highlights the value of a multidisciplinary approach involving oncologists, gastroenterologists, and supportive care teams to optimize outcomes. Future studies should focus on identifying predictive biomarkers for gastrointestinal toxicity to guide safer chemotherapy regimens.
